# Antimicrobial Peptide Identified via Machine Learning Presents Both Potent Antibacterial Properties and Low Toxicity toward Human Cells

**DOI:** 10.3390/microorganisms12081682

**Published:** 2024-08-15

**Authors:** Qifei Wang, Junlin Yang, Malcolm Xing, Bingyun Li

**Affiliations:** 1 Department of Orthopaedics, School of Medicine, West Virginia University, Morgantown, WV 26506, USA; wangqifeicn@163.com; 2 Department of Orthopaedics, The Second Affiliated Hospital of Anhui Medical University, Hefei 230601, China; 3 Spine Center, Xin Hua Hospital Affiliated to Shanghai Jiao Tong University School of Medicine, Shanghai 200082, China; yjunlin@126.com; 4 Department of Mechanical Engineering, University of Manitoba, Winnipeg, MB R3T2N2, Canada; malcolm.xing@umanitoba.ca

**Keywords:** antimicrobial peptide, viability, cytotoxicity, infection, orthopedic

## Abstract

Preventing infection is a critical clinical challenge; however, the extensive use of antibiotics has resulted in remarkably increased antibiotic resistance. A variety of antibiotic alternatives including antimicrobial peptides (AMPs) have been studied. Unfortunately, like most conventional antibiotics, most current AMPs have shown significantly high toxicity toward the host, and therefore induce compromised host responses that may lead to negative clinical outcomes such as delayed wound healing. In this study, one of the AMPs with a short length of nine amino acids was first identified via machine learning to present potentially low cytotoxicity, and then synthesized and validated in vitro against both bacteria and mammalian cells. It was found that this short AMP presented strong and fast-acting antimicrobial properties against bacteria like *Staphylococcus aureus*, one of the most common bacteria clinically, and it targeted and depolarized bacterial membranes. This AMP also demonstrated significantly lower (e.g., 30%) toxicity toward mammalian cells like osteoblasts, which are important cells for new bone formation, compared to conventional antibiotics like gentamicin, vancomycin, rifampin, cefazolin, and fusidic acid at short treatment times (e.g., 2 h). In addition, this short AMP demonstrated relatively low toxicity, similar to osteoblasts, toward an epithelial cell line like BEAS-2B cells.

## 1. Introduction

In the management of surgeries, including orthopedic procedures, preventing infections is one of the major tasks that plays an important role in treatment outcomes [1]. The wide use of implants among various surgeries has significantly increased infection occurrence clinically [2], while the global overuse of antibiotics has led to bacterial antibiotic resistance that has resulted in an increase in ‘superbugs’ like methicillin-resistant *Staphylococcus aureus* (MRSA) [3]. There is an urgent need for drugs that can be used as substitutes for antibiotics.

Since the 1980s, antimicrobial peptides (AMPs), existing in a variety of forms in our lives [4], have emerged as alternatives to conventional antibiotics [5,6,7,8,9]. Natural AMPs have common characteristics; they are relatively short, cationic (+2 to +9), amphiphilic (>50% hydrophobic amino acids or AAs), thermostable (up to 100 °C), etc. [10,11]. AMPs may kill bacteria more rapidly than conventional antibiotics [9,12,13]. Moreover, AMPs could be used as immunomodulatory molecules to regulate the body’s immune function, change the host immune-related gene expression, suppress the inflammatory cytokines induced by lipopolysaccharides, promote wound healing, and enhance angiogenesis. They may also play a bridge role for mononuclear cells, macrophages, and dendritic cells in congenital immune and acquired immune responses [14,15]. However, most AMPs such as LL-37 present high cytotoxicity toward human cells [16,17,18].

Meanwhile, discovering new AMPs with specific properties (e.g., both strong antimicrobial property and low cytotoxicity toward hosts) has been challenging since traditional methods are often time-consuming and costly, involving extensive laboratory testing. However, artificial intelligence, like machine learning, offers a powerful alternative by leveraging large datasets that are available and sophisticated algorithms to potentially predict AMP candidates of desired properties with high accuracy and efficiency. For instance, machine learning was used to identify AMPs via screening about 100,000 peptides from an in silico library with a 94% accuracy [19]. Machine learning was also combined with a genetic algorithm to identify AMPs that are antimicrobial against specific bacteria like *Staphylococcus epidermidis* [20]. Such approaches may accelerate the discovery process, reduce costs, and enable the identification of novel AMPs that might be overlooked by conventional methods.

In this study, an AMP with a nine AA sequence of KRWWKWWRR (referred to as HHC36 in the literature) was first selected via machine learning, and then synthesized and tested in vitro against bacteria and mammalian cells. We hypothesized that HHC36 identified via machine learning has high in vitro antimicrobial activity against bacteria like *Staphylococcus aureus* (*S. aureus*) and low toxicity toward mammalian cells like osteoblasts.

## 2. Materials and Methods

### 2.1. Materials

The HHC36 to be assessed in this study was synthesized by CPC Scientific Inc. (Sunnyvale, CA, USA), and its sequence is Lys-Arg-Trp-Trp-Lys-Trp-Trp-Arg-Arg-NH_2_ (KRWWKWWRR-NH_2_) with an MW of 1487.8 and a purity of 98.2% (determined by high-performance liquid chromatography). Conventional antibiotics (i.e., gentamicin, daptomycin, vancomycin, rifampin, cefazolin, and fusidic acid) were purchased from Millipore Sigma (St. Louis, MO, USA). Propidium iodide (PI) was obtained from MP Biomedicals (Solon, OH, USA). Dimethyl sulfoxide (DMSO) was from Thermo Fisher Scientific (Waltham, MA, USA). DiBAC4(3) bis-(1,3-dibutylbarbituric acid) trimethine oxonol was obtained from ANASPEC (Fremont, CA, USA). The 3-(4,5-dimetyl-2-tiazolyl)-2,5-diphenyl-2Htetrazolium bromide or MTT assay was obtained from Biotium (Fremont, CA, USA). Tryptic soy broth (TSB) was purchased from Becton, Dickinson, and Company (Sparks, MD, USA) and sheep blood agar plates from Remel (Thermo Fisher Scientific, San Diego, CA, USA). Human osteoblast cells hFOB 1.19 (ATCC CRL-3602) and human lung bronchial epithelial BEAS-2B cells (ATCC CRL-9609) were obtained from American Type Culture Collection (ATCC, Manassas, VA, USA). Dulbecco’s Modified Eagle Medium (DMEM), phosphate buffered saline (PBS), fetal bovine serum (FBS), and Triton X-100 were purchased from Thermo Fisher Scientific. *S. aureus* was from a patient’s wound at Ruby Memorial Hospital (Morgantown, WV, USA). *S. aureus* was studied because it is a major bacterium that leads to numerous infections in clinics including orthopedic infections.

### 2.2. Antimicrobial Experiments

The minimal inhibitory concentration (MIC) [21] of HHC36 was tested using our established protocol [22] and compared to daptomycin and gentamicin. In brief, *S. aureus* at the exponential phase was prepared in Mueller Hinton broth to have ~10^5^ colony-forming units (CFU)/mL, and antimicrobial solutions of various concentrations of HHC36, daptomycin, and gentamicin were prepared with twofold dilutions. Next, 90 µL of *S. aureus* and 10 µL of one of the antimicrobial solutions were added together to a well in a 96-well plate. After incubating overnight at 37 °C, the plate was read at 600 nm and recorded. The highest concentration of a specific antimicrobial agent that presented no bacterial growth is defined as the MIC of that specific antimicrobial agent.

The antimicrobial effect of the HHC36 and antibiotics was also tested by a survival assay via counting the residual number of CFU in triplicate experiments. *S. aureus* was grown in sheep blood agar and cultured overnight at 37 °C in an incubator under aerobic conditions. Three colonies of *S. aureus* were added to 5 mL of TSB and incubated at 37 °C for 16 h. Next, 100 μL of the bacteria was added to 20 mL of TSB and incubated at 37 °C for 2 h. The bacterial suspension was then diluted with PBS+ (with Ca^2+^ and Mg^2+^) and was adjusted to a final concentration of 3 × 10^5^ CFU/mL using a spectrophotometer (Thermo Fisher Scientific).

The concentrations of HHC36 tested were 3, 30, 100, 200, and 300 μM, and the concentrations of antibiotics (gentamicin, vancomycin, rifampin, cefazolin, and fusidic acid) were 200 μM and 2 mM. The experiments were conducted by adding *S. aureus* (3 × 10^5^ CFU/mL) and various concentrations of HHC36 or one of the antibiotics to a total volume of 1 mL. The treated samples and control (without the addition of HHC36 and antibiotics) were placed in a reciprocal shaking bath and kept at 37 °C for 30 min. Dilutions of 10^−2^, 10^−3^, and 10^−4^ were carried out with PBS+, and the drop plate method [13] was applied to count the viable bacteria. For the drop plate method, a blood agar plate was separated into six parts and a bacterial suspension of 20 μL was then placed on each part, dried, and then the plate was inverted and placed in the incubator at 37 °C for 24 h. Triplicate samples were run and the CFU were determined and percent bacterial killing was calculated.

Kinetic studies were conducted for HHC36 at 200 μM and antibiotics at 2 mM at various time intervals (5, 10, 15, and 30 min, or 30, 60, 120, and 360 min, respectively). The kinetics experiments were conducted by adding log-phase bacteria (3 × 10^5^ CFU/mL) and the HHC36 or one of the antibiotics in a total sample volume of 1 mL. After incubating for a certain time, samples (control and treated ones) were diluted and plated on 5% sheep blood agar plates, and the CFU were subsequently determined.

### 2.3. Bacterial Membrane Permeabilization and Depolarization

Membrane permeabilization and membrane depolarization experiments were carried out according to a reported protocol [23]. For permeabilization, *S. aureus* at the exponential phase was prepared and diluted to OD600 0.11 with TSB. Antimicrobial solutions of HHC36, daptomycin, and gentamicin at 2× MIC were prepared and used. The 20 mM PI was prepared in DMSO in the dark, and this stock solution was diluted to obtain 1 mM PI with deionized water. A volume of 10 μL of an antimicrobial solution, 2 μL of PI (1 mM), and 88 μL of *S. aureus* (total of 100 µL) were added to a 96-well plate per well and incubated at 37 °C with continuous shaking. The samples were read at 10, 30, 50, 70, 90, and 110 min with excitation and emission wavelengths at 584 nm and 620 nm, respectively. For the depolarization experiments, *S. aureus* was grown in TSB to exponential phase, washed with PBS, re-suspended in PBS that contained glucose (25 mM), and incubated at 37 °C for 15 min. Next, DiBAC4 (3) bis-(1,3-dibutylbarbituric acid) trimethine oxonol at 500 nM was added and gently mixed via vortexing. Aliquots (90 µL per well) of the bacterial solution were then placed into 96-well plates, and their fluorescence was read at excitation and emission wavelengths of 485 nm and 520 nm, respectively. Then, 10 µL of the antimicrobial solution (2× MIC) was added and fluorescence readings were obtained every 5 min up to 45 min. Triton X-100 (0.1%) was included as a positive control.

### 2.4. Cell Viability Experiments

Human osteoblast cells and BEAS-2B cells were used, and their viability was measured using the MTT assay [24,25,26]. The cells were cultured in a medium consisting of DMEM, including 10% FBS, 1% nonessential AAs, G418 (geneticin), and 1% penicillin/streptomycin. The culture medium was replaced at two-day intervals and kept at 37 °C in a 95% humidified atmosphere with 5% CO_2_. A cell suspension (3 × 10^5^ cells/mL) was prepared, and 100 μL of the suspension was added to each well in a 96-well plate to obtain 3 × 10^4^ cells/well. The plate was incubated for 24 h to achieve cell adherence. The HHC36 (30, 100, 200, 300, and 500 μM) or antibiotic (200 μM and 2 mM) treatment was then added to each well and incubated. Triplicate samples were assessed, and controls (without HHC36 treatment) were run at the same time. After culturing for various times (e.g., 2, 6, 12, and 24 h), the wells were washed twice with PBS and 100 μL of unsupplemented media (without FBS or penicillin/streptomycin) with 10 μL of MTT reagent were added to each well and incubated for 2 h. The formation of formazan crystals was assessed under a microscope and 150 μL of DMSO was then added to each well. The plates were shaken for 15 min, and the absorbance at 570 nm was measured and recorded. The same MTT assay procedure was applied to assess the effect of HHC36 on the viability of BEAS-2B cells. The absorbance of each sample was compared with a control group and expressed as a percentage as compared to the control.

### 2.5. Peptide Selection from AMP Database

The APD3 database, one of the best-known AMP databases with experimentally validated AMPs found in nature and in the literature [27,28], was used to select AMPs with potentially high antimicrobial and low cytotoxicity properties. The 3940 available peptides in the APD3 database [29] were screened to obtain peptide sequences with anti-Gram-positive, anti-Gram-negative, anti-MRSA activities, and ≤20 AAs in length; short peptides are economically beneficial in their synthesis. Next, the obtained peptide sequences were further screened to obtain the sequence that had high antimicrobial probability (>90%), low toxicity probability (<50%), and the lowest hydrophobicity; the lower the hydrophobicity, the lower the hemolytic activity [30,31]. The APD3 database was used to obtain the antimicrobial activity probability [32], which was predicted using deep neural network with the prediction based on convolutional and long short-term memory (LSTM) layers [33]; Antimicrobial Peptide Scanner vr.2 [32] was used. CSM-Toxin, an in silico protein toxicity classifier [34], was applied to obtain the cytotoxicity probability, which was predicted with a deep learning model using convolutional neural networks where bidirectional gated recurrent units (BiGRU) instead of LSTM layers were applied; the hydrophobicity was also calculated [35]. The CSM-Toxin classifier also provides the hydrophobicity data calculated by adding the hydrophobicity of each amino acid and dividing by the number of amino acids in the peptide sequence [36].

### 2.6. Statistical Analysis

Data are expressed as mean ± standard deviation and were analyzed using JMP-V17 statistical software. To compare the data from any two groups, a *t*-test was conducted, while among three groups, an analysis of variance (ANOVA) followed by Tukey’s honestly significant difference test was carried out to determine significance. *p* < 0.05 was considered statistically significant.

## 3. Results

### 3.1. Peptide Selection from AMP Database

**Figure 1:**
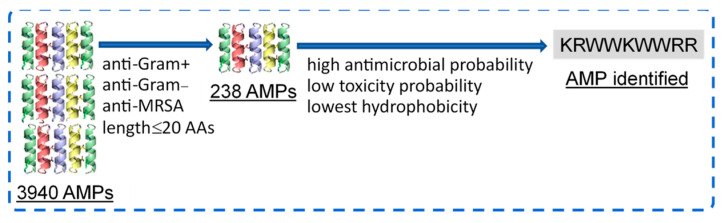
Selection of an AMP with high antimicrobial and low cytotoxicity probabilities via machine learning.

From the 3940 peptides in the APD3 database, 238 AMPs were identified to present anti-Gram-positive, anti-Gram-negative, and anti-MRSA activities with lengths ≤ 20 AAs (Figure 1). Next, the peptide sequence KRWWKWWRR (referred to as HHC36 in the literature) was further identified to have high antimicrobial probability (1.00), low toxicity probability (0.30), and the lowest hydrophobicity (−2.77). The antimicrobial and cytotoxicity properties of KRWWKWWRR identified were further validated via experiments, as detailed below.

### 3.2. Antimicrobial Activity

It was found that HHC36 had a much higher MIC compared to daptomycin, vancomycin, and gentamicin. The MICs of HHC36, daptomycin, vancomycin, and gentamicin were 16 µM (23.80 µg/mL), 0.5 µM (0.81 µg/mL), 1.0 µM (1.45 µg/mL), and 0.25 µM (0.12 µg/mL), respectively.

**Figure 2:**
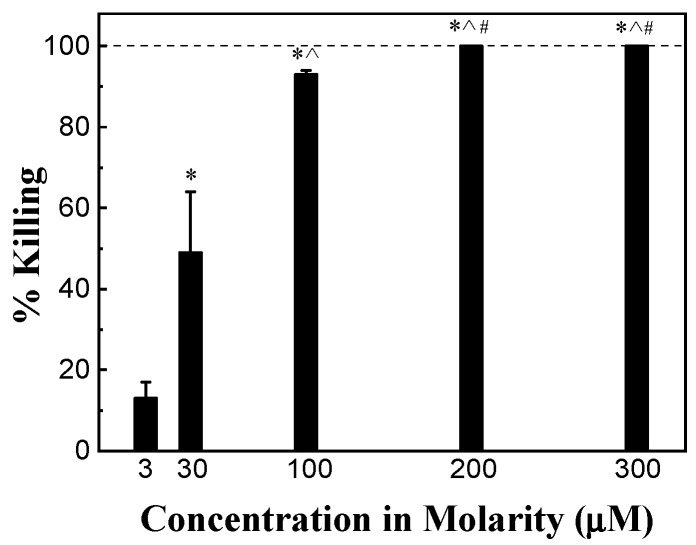
Percent killing of *S. aureus* by HHC36 at 30 min. * *p* < 0.05 compared to 3 µM treatment; ^ *p* < 0.05 compared to 30 µM treatment; and ^#^ *p* < 0.05 compared to 100 µM treatment.

The antimicrobial activity of HHC36 of different concentrations varying from 3 μM to 300 μM (3, 30, 100, 200, 300 μM) was tested. After 30 min, the percent killing of *S. aureus* was about 15% at 3 μM, and increased sharply and significantly to approximately 50% at 30 μM and over 90% at 100 μM. An HHC36 concentration of 200 μM and above (i.e., 300 μM) achieved 100% killing (Figure 2).

**Figure 3:**
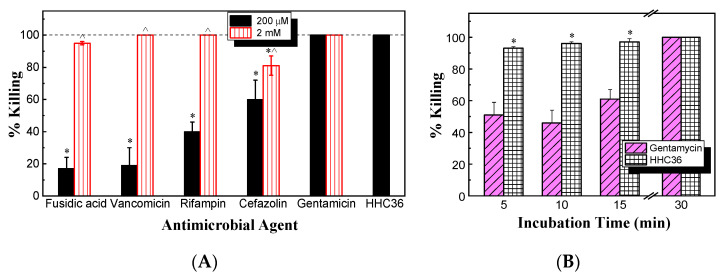
(**A**) Percent killing of *S. aureus* by HHC36 and multiple conventional antibiotics for 30 min. * *p* < 0.05 compared to HHC36 treatment; ^ *p* < 0.05 compared to the 200 µM treatment of the same antibiotic. (**B**) Bacterial killing kinetics against *S. aureus* of HHC36 and gentamicin at 200 µM. * *p* < 0.05 compared to gentamicin at the same treatment time.

Next, the antimicrobial activity against *S. aureus* between HHC36 and conventional antibiotics (gentamicin, vancomycin, rifampin, cefazolin, and fusidic acid) was compared. After culturing for 30 min at the same concentration of 200 μM, the results (Figure 3A) show that vancomycin, rifampin, cefazolin, and fusidic acid all had significantly lower percent killing compared to HHC36. HHC36 had 100% killing, while fusidic acid and vancomycin presented only about 20% killing of *S. aureus*; rifampin and cefazolin about 40% and 60% killing, respectively; and gentamicin achieved 100% killing. When the concentration increased ten times to 2 mM, the percent killing of cefazolin was significantly lower compared to that of HHC36, and vancomycin, rifampin, cefazolin, and fusidic acid showed significantly higher bacterial killing compared to their respective percent killing at 200 μM (Figure 3A).

**Figure 4:**
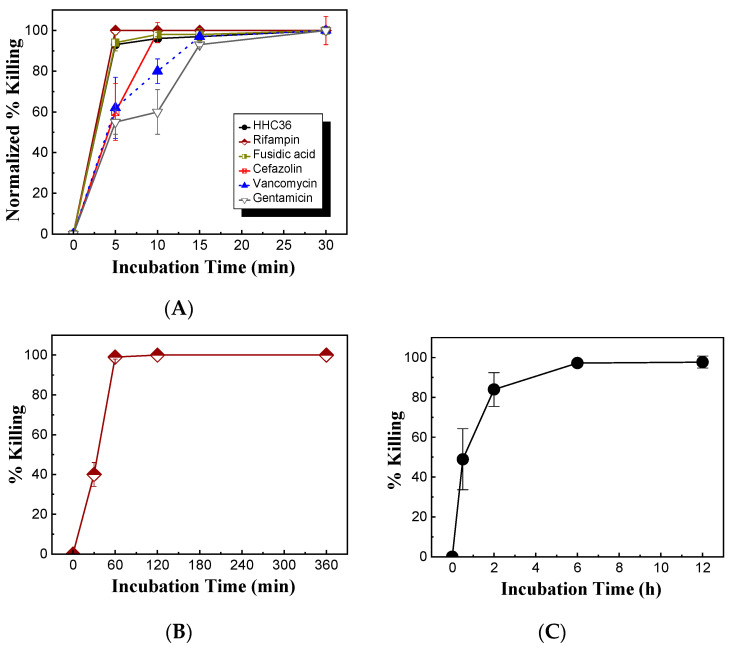
(**A**) Comparison of bacterial killing of HHC36 and conventional antibiotics at various treatment times. The concentrations of antibiotics were 2 mM, and the concentration of HHC36 was 200 µM. (**B**) Bacterial killing of rifampin of 200 µM at various treatment times. (**C**) Bacterial killing of HHC36 at a low concentration (i.e., 30 µM) at various treatment times.

Meanwhile, the bacterial killing kinetics of HHC36 were investigated and compared with those of gentamicin at the concentration of 200 μM (Figure 3B). At 200 μM, HHC36 eliminated more than 90% of *S. aureus* within 5 min and killed all the bacteria within 30 min. In contrast, gentamicin had much slower kinetics and achieved about 60% bacterial killing within 15 min and 100% killing at 30 min. At 5, 10, and 15 min, the bacterial killing by gentamicin was significantly lower compared to HHC36 (Figure 3B). At a much higher concentration (i.e., 2 mM), gentamicin, vancomycin, and cefazolin had much lower bacterial killing compared to HHC36, rifampin, and fusidic acid at 5 min; gentamicin and vancomycin also showed much lower bacterial killing at 10 min compared to the other treatments (Figure 4A). Antibiotics like rifampin took a long time for bacterial killing; rifampin at 200 µM reached about 40% killing at 30 min and 100% bacterial killing at 60 min (Figure 4B). Similarly, lower concentrations of HHC36 (e.g., 30 µM) took a long time (e.g., hours) to achieve 100% bacterial killing (Figure 4C).

### 3.3. Bacterial Membrane Permeabilization and Depolarization

**Figure 5:**
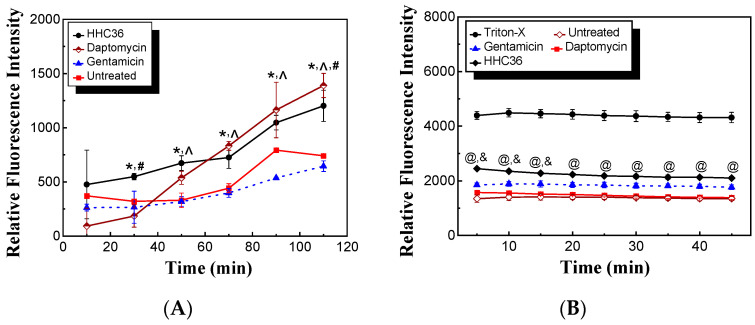
(**A**) Permeabilization of *S. aureus* membrane treated with HHC36, daptomycin, gentamicin, and control (untreated) at 2× MIC. * *p* < 0.05 HHC36 vs. untreated and gentamicin treatment at the same time period; ^ *p* < 0.05 daptomycin treatment vs. untreated and gentamicin treatment at the same time period. ^#^ *p* < 0.05 HHC36 vs. daptomycin treatment. (**B**) Depolarization of *S. aureus* membrane treated with HHC36, daptomycin, gentamicin, Triton-X100, and untreated. The concentrations of HHC36, daptomycin, and gentamicin were 2× MIC. Triton-X100 of 0.1% was used. ^@^ *p* < 0.005 HHC36 vs. Triton-X100, daptomycin, and untreated; ^&^ *p* < 0.05 HHC36 vs. gentamicin.

AMPs may target and depolarize bacterial membranes. To determine if HHC36 targets and depolarizes *S. aureus* membranes, we carried out membrane permeabilization and depolarization experiments in the presence of PI. HHC36 treatment presented significantly higher relative fluorescence intensity compared to the untreated samples and gentamicin treated samples at 30 min and longer, and daptomycin had significantly higher relative fluorescence intensity compared to the untreated samples and gentamicin treated samples at 50 min and longer (Figure 5A). Moreover, HHC36 had significantly higher relative fluorescence intensity at 30 min, significantly lower at 110 min, and no significant differences at other time points compared to the daptomycin treatment (Figure 5A). Gentamicin, which inhibits protein synthesis, was used as a negative control while daptomycin, which targets bacterial membranes, was used as a positive control in similar studies [23]. As to the membrane depolarization experiments, HHC36 presented significantly higher relative fluorescence intensity compared to the untreated samples and the treated samples with daptomycin at all time points and with gentamicin treatment at 5, 10, and 15 min; meanwhile, HHC36 had significantly lower fluorescence intensity at all time points compared to the samples treated with Triton-X100, which is known to be a strong membrane depolarization agent (Figure 5B). No significant differences were observed between the daptomycin treatment and the untreated samples.

### 3.4. Cell Viability

**Figure 6:**
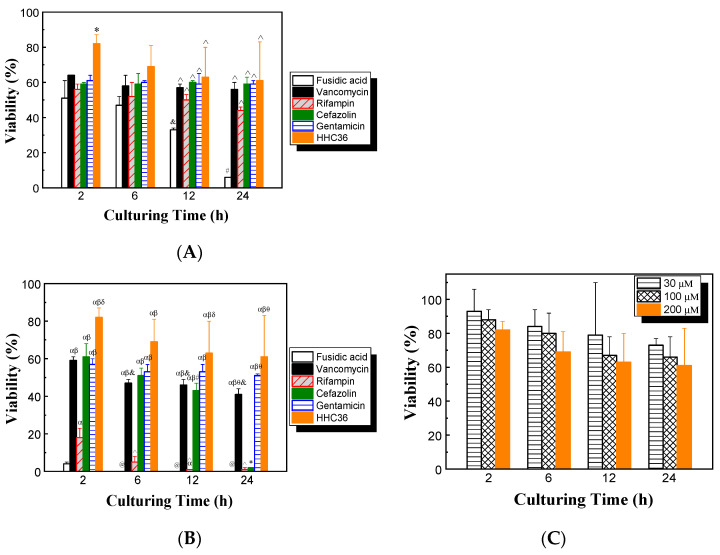
(**A**) Effects of different antimicrobial agents on the viability of osteoblasts; concentrations of all antimicrobial agents were 200 µM. * *p* < 0.05 compared to all other antibiotic treatments at 2 h; ^ *p* < 0.05 compared to fusidic acid treatment at the same culturing time; ^&^ *p* < 0.05 compared to fusidic acid treatment at 2 h; ^#^ *p* < 0.05 compared to fusidic acid treatment at 2, 6, and 12 h. (**B**) Effects of different antimicrobial agents on the viability of osteoblasts; concentrations of all conventional antibiotics were 2 mM and the concentration of HHC36 was 200 µM. ^α^ *p* < 0.05 compared to fusidic acid treatment at the same culturing time; ^β^ *p* < 0.05 compared to rifampin treatment at the same culturing time; ^δ^ *p* < 0.05 compared to vancomycin, cefazolin, and gentamicin treatments at the same culturing time; ^θ^ *p* < 0.05 compared to cefazolin treatment at the same culturing time; * *p* < 0.05 compared to cefazolin treatments at 2, 6, and 12 h; ^@^ *p* < 0.05 compared to fusidic acid treatment at 2 h; ^&,^^ *p* < 0.05 compared to vancomycin or rifampin treatment at 2 h, respectively; ^#^ *p* < 0.05 compared to cefazolin treatment at 2 h. (**C**) Effects of culturing time and HHC36 concentration on the viability of osteoblasts.

The cytotoxicities of HHC36 and conventional antibiotics on mammalian cells were determined. The results show that with treatment of HHC36 at 200 μM, the viability of human osteoblast cells was over 80% at 2 h, then decreased to about 70% at 6 h and 60% at 12 and 24 h (Figure 6A). At 2 h, HHC36 showed significantly higher (30% higher) osteoblast cell viability compared to all conventional antibiotic treatments studied. HHC36 treatment also showed significantly higher osteoblast cell viability compared to fusidic acid treatment at 12 and 24 h. Fusidic acid showed the lowest osteoblast cell viability among all treatments at all time points studied, and the differences were significant at 12 and 24 h. Fusidic acid treatment also presented significantly reduced osteoblast cell viability at 12 h compared to 2 h and at 24 h compared to 2, 6, and 12 h; fusidic acid treatment had only 6% osteoblast cell viability at 24 h (Figure 6A).

The effects of high-concentration antimicrobial treatments on osteoblast cell viability were also assessed. Concentrations of 2 mM were chosen for all antibiotics, since it was shown that the antibiotics tested at this concentration had 100% or close to 100% bacterial killing (Figure 3A), while HHC36 had 100% bacterial killing at 200 µM (Figure 2). The results show that, at 2 mM, both fusidic acid and rifampin were toxic to osteoblast cells, since the osteoblast cell viability was lower than 20% at all time points tested. At 12 and 24 h, almost all of the osteoblast cells had been killed (Figure 6B). At 24 h, cefazolin also showed low osteoblast cell viability. Gentamicin and vancomycin had significantly higher osteoblast cell viability at all time points tested compared to rifampin and fusidic acid (Figure 6B).

The effects of culturing time and HHC36 concentration on the viability of osteoblast cells were determined. Overall, the average osteoblast cell viability decreased with increasing HHC36 concentration and culturing time, while the changes were not statistically significant (Figure 6C). For instance, at 30 μM, the average osteoblast cell viabilities decreased from 93% at 2h to 84%, 79%, and 73% at 6, 12, and 24 h, respectively; the average viabilities were 93%, 88%, and 82% for 30, 100, and 200 µM, respectively.

**Figure 7:**
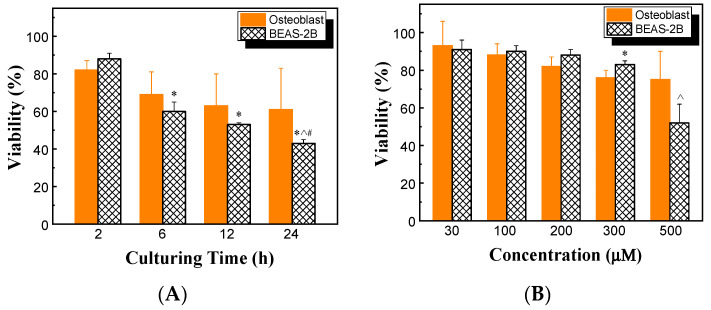
(**A**) Viabilities of osteoblast and BEAS-2B cells at 200 µM HHC36. * *p* < 0.05 compared to BEAS-2B at 2 h; ^ *p* < 0.05 compared to BEAS-2B at 6 h; ^#^ *p* < 0.05 compared to BEAS-2B at 12 h. (**B**) Viabilities of osteoblast and BEAS-2B cells for HHC36 of various concentrations at 2 h culturing time. * *p* < 0.05 compared to osteoblast at 300 µM; ^ *p* < 0.05 compared to BEAS-2B at 30, 100, 200, and 300 µM.

The effects of HHC36 concentration and culturing time on the viability of BEAS-2B cells were also determined and compared with those of osteoblast cells. At 200 μM HHC36, the viability of BEAS-2B cells decreased significantly with increasing culturing time, while no significant differences were observed between BEAS-2B and osteoblast cells (Figure 7A). BEAS-2B had significantly lower viability at 500 µM compared to the other concentrations studied (i.e., 30, 100, 200, and 300 µM). Among the treatments of varying concentrations of HHC36, the viabilities of BEAS-2B and osteoblast cells were similar at concentrations at or below 200 µM, and BEAS-2B had a significantly higher viability at 300 µM but a significantly lower viability at 500 µM (Figure 7B).

## 4. Discussion

Throughout the history of utilizing antimicrobial agents, there has been a challenge that has not been addressed where most, if not all, antimicrobial agents have resulted in reduced host cell viability, thereby compromising host responses. In the field of orthopedics, this issue is reflected as the inhibition of osteogenesis, which, for instance, may lead to delayed unions or non-unions of fractures. Therefore, identifying and developing antimicrobial agents that not only achieve strong antimicrobial properties but also present little toxicity toward mammalian cells are clinically important. In this study, an AMP with the sequence of KRWWKWWRR was identified, synthesized, and tested, aiming to determine if HHC36 has strong antimicrobial properties along with low toxicity toward human cells.

Virtually all species of life produce AMPs, which play an important role in the host defense system against microorganisms. Compared to conventional antibiotics, AMPs are less likely to provoke resistance due to their multiple targeting and interaction with fundamental physiological structures of pathogens [4,37,38]. Multiple AMPs including enfuvirtide and bacitracin have been approved by the Food and Drug Administration for treating infectious diseases [12]. In the literature, numerous studies have demonstrated that AMPs have broad spectrum antibacterial activities [39,40,41]. In general, AMPs have shown fairly effective in vitro antimicrobial activity, and many AMPs have shown the ability to eliminate both Gram-positive and Gram-negative bacteria. AMPs can present their antibacterial activities for different targets, of which the most obvious feature is the targeting of the membrane structure. Bacterial membranes are negatively charged (due to the presence of highly electronegative chemical groups from the phospholipids and lipopolysaccharides on the membrane surface), and AMPs tend to selectively attack bacteria instead of mammalian cells due to the cationic characteristics of AMPs. However, most current AMPs present significantly high toxicity toward human cells, therefore leading to negative clinical outcomes such as delayed wound healing. It is reasonable, though not yet well understood, that their toxic effects are peptide sequence or chemical structure dependent; AMPs with certain AA sequences may have limited toxicity toward mammalian cells [42].

In this study, both the antimicrobial and cytotoxicity properties (against mammalian cells) of one of the AMPs (i.e., HHC36) were investigated. The data showed that, compared with commonly used conventional antibiotics, HHC36 was effective and prompt (Figure 2 and Figure 3) at eliminating bacteria like *S. aureus*. HHC36 was remarkably potent in killing more than 90% of *S. aureus* at 100 μM within 30 min and within 5 min at 200 μM. HHC36 at 200 µM resulted in 100% bacterial killing within 30 min, while in comparison, the bacterial killing of vancomycin, rifampin, cefazolin, and fusidic acid, were at 60% or lower. Gentamicin presented 100% killing at 200 µM within 30 min, while its bacterial percentage killing at 5, 10, and 15 min was significantly lower compared to that of HHC36. Specifically, both HHC36 and gentamicin reached 100% bacterial killing within 30 min at 200 µM (Figure 3A), while HHC36 had much faster kinetics (Figure 3B and Figure 4A). These differences are associated with our findings that HHC36 showed significantly higher permeabilization and depolarization of *S. aureus* membranes compared to gentamicin, and like daptomycin, HHC36 targeted and depolarized *S. aureus* membranes, and it presented significantly higher depolarization of *S. aureus* membranes compared to daptomycin (Figure 5).

Moreover, the findings showed that HHC36 presented significantly higher osteoblast cell viability at short culturing times (e.g., 2 h) compared to all the conventional antibiotics studied (Figure 6A). To achieve the same or similar bacterial percent killing as HHC36, much higher (10 times or more) concentrations of antibiotics like vancomycin, rifampin, cefazolin, and fusidic acid were needed (Figure 3A), while these high concentrations resulted in significantly lower osteoblast cell viability (Figure 6B). HHC36 also showed similar toxicity effects on another mammalian cell type (i.e., BEAS-2B) since no significant differences were observed between osteoblast and BEAS-2B cells at various culturing times at 200 µM (Figure 7A). However, a significant difference in viability was observed at 300 µM (Figure 7B), suggesting the viability effect was concentration dependent.

Therefore, compared to the commonly used antibiotics tested in this study, HHC36 seems to present higher in vitro antimicrobial properties and lower toxicity toward mammalian cells, and it may have potential as a better alternative to the current antibiotics, although further studies are warranted.

In addition, it is important to note that, in the literature [43,44,45,46], it was evident that HHC36 is one of the short AMPs with strong antimicrobial properties, consistent with our findings from the selection of HHC via machine learning in this study. HHC-36, presenting MICs of 0.3–11 µM against multidrug-resistant *Pseudomonas aeruginosa* and MRSA, showed clear superiority to several other AMPs including Bac2A (RLARIVVIRVAR) with corresponding MICs of 3–192 µM and MX-226 (ILRWPWWPWR) with MICs of 10–76 µM [43]; the latter has demonstrated efficacy in limiting catheter infections in phase IIIa clinical trials [47]. In the same study, HHC-36 also presented broader and, in most cases, more potent activity compared to a few widely used clinical antibiotics like tobramycin, ciprofloxacin, imipenem, and ceftazidime [43]. However, there were no reports in the literature comparing the cytotoxicity properties of HHC36 with other antimicrobial agents.

One limitation of this study is that only one strain of bacteria was tested, while the pathogenic microorganisms faced clinically are more complex. Also, in recent decades, intracellular bacteria and small colony variants (SCVs) are believed to play an important role in chronic and recurrent infections, and the antimicrobial effects of HHC36 against intracellular bacteria and SCVs are unknown. This study established solid in vitro data on the antimicrobial properties of HHC36 and, more importantly, it showed that HHC36 presented low toxicity toward mammalian cells. However, its in vivo toxicity and its effect on wound healing, etc., are unclear and may be investigated in the future using models like open fracture infection rat models [48,49,50]. One of the features used to select the peptide sequences was low hydrophobicity, leading to limited hemolytic activity [30,31], which may also be examined in future studies.

## 5. Conclusions

A short AMP HHC36 with strong antimicrobial and low cytotoxicity properties was selected via machine learning, and then validated via cell culture studies. In this study, *S. aureus*, one of the most common causes of bacterial infections, was treated with HHC36 and conventional antibiotics for antimicrobial tests; human osteoblast and BEAS-2B cells were also treated with these antimicrobial agents for mammalian cell toxicity assessment. HHC36 was found to have rapid and robust bacterial killing against *S. aureus*, and also presented relatively low toxicity toward the human cells studied. HHC36 at 200 μM eliminated 93% (40% more than gentamicin) of the bacteria within 5 min, and had a viability of 82% (20% or more compared to the conventional antibiotic treatments) for osteoblast cells and 88% for BEAS-2B cells at 2 h. Therefore, one can conclude that HHC36 had rapid and remarkable in vitro antimicrobial properties toward *S. aureus*, targeted and depolarized *S. aureus* membranes, and presented relatively low toxicity toward mammalian cells. In future studies, the antibacterial and toxicity effects of HHC36 against various types of bacteria and human cells may be further examined both in vitro and in vivo.

## Data Availability

The raw data supporting the conclusions of this article will be made available by the authors on request.
